# Immigrants’ use of primary health care services for mental health problems

**DOI:** 10.1186/1472-6963-14-341

**Published:** 2014-08-13

**Authors:** Melanie Straiton, Anne Reneflot, Esperanza Diaz

**Affiliations:** Division of Mental Health, Norwegian Institute of Public Health, Oslo, Norway; Department of Global Public Health and Primary Care, University of Bergen, Bergen, Norway

**Keywords:** Immigrant health, Primary health care, Mental health, Health inequalities

## Abstract

**Background:**

Equity in health care across all social groups is a major goal in health care policy. Immigrants may experience more mental health problems than natives, but we do not know the extent to which they seek help from primary health care services. This study aimed to determine a) the rate immigrants use primary health care services for mental health problems compared with Norwegians and b) the association between length of stay, reason for immigration and service use among immigrants.

**Methods:**

National register data covering all residents in Norway and all consultations with primary health care services were used. We conducted logistic regression analyses to compare Norwegians’ with Polish, Swedish, German, Pakistani and Iraqi immigrants’ odds of having had a consultation for a mental health problem (P-consultation).

**Results:**

After accounting for background variables, all immigrants groups, except Iraqi men had lower odds of a P-consultation than their Norwegian counterparts. A shorter length of stay was associated with lower odds of a P-consultation.

**Conclusions:**

Service use varies by country of origin and patterns are different for men and women. There was some evidence of a possible ‘healthy migrant worker’ effect among the European groups. Together with previous research, our findings however, suggest that Iraqi women and Pakistanis in particular, may experience barriers in accessing care for mental health problems.

## Background

There is a growing body of evidence suggesting that immigrants’ patterns of health care utilisation differ to that of natives [1–3]. Service use is likely to vary depending on the health care system available, the service (general or specialised), the health issue and the immigrant population studied [4]. Differences in health service use may reflect difficulties in accessing health services, differences in actual health status or health care preferences. Reliable, representative data on immigrants’ use of health services is required as a first step towards assessing and understanding differences in equity [5].

Regarding all health problems, differences between immigrants’ and natives’ use of emergency primary health care (EPC) have been documented, though service use among immigrants varies by factors such as age, country background, immigration reason, length of stay and socioeconomic status [2, 6–9]. A recent, national-level Norwegian study found that contact rates at EPC services were lower for immigrants as a whole compared with Norwegians, though higher among specific groups [3]. Findings are also mixed for general practitioner (GP) visits, with some studies finding lower use among immigrants [1, 10] and others higher [2, 11–13]. Although gender differences have also been documented [14], not all studies consider men and women separately. Further, contact rates in relation to mental health problems have not yet been investigated.

In Norway, the health service is publicly funded and available to all citizens and residents, covering regular medical consultations (subject to a small fee), emergency treatment and hospitalisation. GPs play a large role in the diagnosis and management of mental health care, and are gate-keepers to accessing specialised mental health services. This means that majority of individuals who seek professional help will come into contact with primary health care services (PHC). The aim of this study is to determine the rate to which adult immigrants use PHC for mental health problems compared with Norwegians. Using national register data covering all residents in Norway, we focus on the five largest immigrant groups and consider whether differences in service use are explained by a range of demographic variables. We investigate GP and EPC services separately and whether the same patterns are found for both men and women. For immigrants, we also assess the impact of reason for immigration and length of stay on service use.

## Methods

This study uses data from two national registries that were linked together for the year 2008 as part of a larger project looking at immigrants’ health in Norway (Immigrants’ health in Norway). At birth, all Norwegian citizens are given a unique personal identification number (ID number), as are immigrants who stay in Norway for more than 6 months. ID numbers were used to link the registries together on an individual level.

The National Population Register provides information about all residents in Norway. Relevant variables derived from this database include gender, age group (18–25, 26–35, 36–45, 46–55, 56–67), immigration category (native Norwegian or immigrant), country of origin (covers country background over two generations), reason for immigration (recorded since 1990), length of stay in Norway, area of residence (based on proximity to a city or town: urban, semi-urban, semi-rural, rural), marital status (married, never married, previously married) and gross personal income (no income, low, medium or high). Income categories were calculated based on median national income; those with 60% or less of the national median income were classed as low-income and those with 60% above the median were classed as high-level income. The Norwegian Health Economics Administration database (HELFO) contains a record of all patient contacts within PHC in Norway. This includes both consultations with regular GPs and EPC services. Relevant variables derived from this database include: whether a patient had received a psychological diagnosis during a GP consultation (GP P-consultation) or an EPC consultation (EPC P-consultation), the number of consultations with a GP for other reasons (GP non P-consultations), and the number of consultations at EPC services for other reasons (EPC non P-consultations). Diagnoses are made based on the International Classification of Primary Care (ICPC-2). Consultations involving any diagnosis from P01-P99 were classed as P-consultations.

Only Norwegian residents aged 18–67 who have an ID-number are included in the current study. In accordance with Statistics Norway, immigrants are defined here as foreign-born individuals with two foreign-born parents. Native Norwegians are defined as Norwegian-born individuals with two Norwegian-born parents [15].

Ethical approval for the main study has been granted by the Norwegian Data Inspectorate and the Regional committee for Medical and Health Research Ethics as well as the Norwegian Labour Welfare Service and the Norwegian Directorate of Health. The Norwegian Social Science Data Service was responsible for supplying the final anonymous data file.

### Analyses

SPSS version 20.0 was used for analyses. Dependent variables were GP P-consultation (yes/no) and EPC P-consultation (yes/no). Individuals with both a GP and EPC P-consultation appeared in both analyses. Chi-square analyses were used to determine if there was a difference in the rate of P-consultations with GP and P-consultations with EPC services between Norwegians and all immigrants. We then selected out the five largest immigrant groups in Norway (those from Poland, Sweden, Germany, Pakistan and Iraq) and compared the demographic profile of each immigrant group to Norwegians using chi-square analyses and analysis of variance with post-hoc Bonferroni tests. A series of univariate logistic regression analyses were then carried out to investigate the influence of age group, marital status, place of residence, personal income level (20+ years only) and general service use (non-P consultations) on odds of P-consultations. Together with country of origin, these variables were entered into multivariate logistic regression analyses to see if there were significant differences between Norwegians and the various immigrant groups. For immigrants, we also conducted separate logistic regression analyses to investigate the importance of length of stay and reason for immigration on P-consultations. Analyses were conducted separately for men and women.

## Results

### Overall P-consultations

In total, there were 2,962,408 individuals in the dataset, 12.1% of whom were immigrants. Around 9.9% of all immigrants and 12.1% of Norwegians had had a GP consultation involving a P-diagnosis (GP P-consultation). For EPC services (EPC P-consultation), these figures were 0.6% and 0.7% for immigrants and Norwegians respectively. Chi-square analyses suggested that these differences were significant (GP: *X*2 = 1504.60, df = 1, p < 0.001; EPC: *X*2 = 143.56, df = 1, p < 0.001). A significantly higher percentage of women than men had had a GP P-consultation (women: 14.5%; men: 9.2% (*X*2 = 19866.51, df = 1, p < 0.001)), but there were no differences for EPC P-consultations (0.7% for both men and women).

Some individuals had had both GP and EPC consultations, giving an overall total of 10.1% of immigrants and 12.3% of Norwegians with at least one P-consultation in 2008. Of those who had had at least one P-consultation, 94.2% had exclusively used a GP for P-consultations, 1.8% had exclusively used EPC services and 4.0% had used both.

### Immigrant groups

The five biggest immigrant groups in Norway account for 30.3% of the total immigrant population and represent various different cultures, reasons for and timing of immigration to Norway. Table 1 provides descriptive background information on each group together with native Norwegians. There are fewer women in the Polish, German and Iraqi immigrant groups compared with natives. Immigrants are younger than Norwegians and, with the exception of Swedes and Germans, are more likely to have no/low level of income. They are also more likely to live in urban areas and, with the exception of Swedes, are more likely to be married.

**Table 1:** **Demographic information for Norwegians and the five biggest immigrant groups in Norway**

	Norway (n = 2604757)	Poland (n = 37669)	Sweden (n = 24656)	Germany (n = 15772)	Pakistan (n = 15067)	Iraq (n = 15053)
% women	49.2%	29.6%*	49.6%	43.7%*	48.6%	40.4%*
% married	45.1%	62.7%*	35.5%*	47.8%*	79.2%*	59.8%*
% living in urban area	65.1%	77.2%*	82.3%*	69.6%*	98.6%*	82.7%*
% with no/low personal income^1^	30.8%	38.5%*	28.9%*	31.1%	61.9%*	63.6%*
Mean age in years (sd)	42.70 (13.96)	36.40* (9.95)	36.40* (13.13)	40.66* (11.81)	40.18* (12.45)	34.72* (10.64)
Reason for immigration^2^						
% for work	-	80.2%	0.3%	67.5%	3.9%	0.4%
% for family	-	17.4%	0.2%	22.2%	85.5%	35.1%
% for protection	-	0.3%	0.0%	0.4%	4.5%	63.3%
% for other/reason not given	-	2.1%	99.5%^4^	9.9%	6.1%	1.2%
Mean length of stay in years (sd)	-	3.53 (6.82)	12.03 (12.52)	9.25 (12.69)	19.35 (11.21)	7.86 (4.81)
% with at least one P-consultation^3^						
Overall	12.3%	3.5%*	9.7%*	6.4%*	11.9%	16.3%*

^1^includes only those aged 20+ years.

^2^excludes those migrating prior to 1990 as this information was not previously recorded.

^3^unadjusted rates – includes both GP and EPC consultations.

^4^Scandinavian immigrants are not required to report reason for migration.

*indicates a significant difference compared to Norwegians (p < 0.05).

Table 1 also displays the overall percentage in each group who had had at least one P-consultation. Compared with Norwegians, Polish, Swedish and German immigrants are less likely to have had a P-consultation, Iraqi immigrants are more likely and there is no difference for Pakistani immigrants.

### Variables associated with P-consultations

Table 2 shows how various background variables relate to GP and EPC P-consultations after adjusting for age-group. Relationships are similar for both men and women. Odds for a GP P-consultation peak amongst middle-aged adults. Odds were higher for non-married individuals and those living in urban areas. Visiting a GP for non-P related reasons was positively associated with having had a GP P-consultation while personal income (20+ years) was negatively related.

**Table 2:** **Age-adjusted odds ratios (OR) and confidence intervals (CI) for GP and EPC P-consultations for men and women by background variables**

	For GP consultation		For EPC consultation	
	Men OR (95% CI)	Women OR (95% CI)	Men OR (95% CI)	Women OR (95% CI)
**Age group**				
18-25	1.00	1.00	1.00	1.00
26-35	1.31 (1.28-1.34)*	1.31 (1.29-1.33)*	0.91 (0.86-0.97)*	0.63 (0.59-0.67)*
36-45	1.32 (1.29-1.35)*	1.48 (1.46-1.51)*	0.73 (0.69-0.78)*	0.62 (0.58-0.65)*
46-55	1.29 (1.27-1.32)*	1.42 (1.40-1.45)*	0.70 (0.66-0.75)	0.58 (0.55-0.62)*
56-67	1.08 (1.06-1.10)*	1.12 (1.10-1.14)*	0.48 (0.45-0.51)*	0.39 (0.36-0.41)*
**Marital status**				
Married	1.00	1.00	1.00	1.00
Never married	1.92 (1.90-1.95)*	1.48 (1.46-1.50)*	4.57 (4.28-4.87)*	2.66 (2.50-2.83)*
Previously married	2.41 (2.37-2.45)*	2.28 (2.25-2.31)*	5.61 (5.24-6.01)*	4.09 (3.85-4.34)*
**Place of residence**				
Urban	1.00	1.00	1.00	1.00
Suburban	0.96 (0.95-0.98)*	0.95 (0.93-0.96)*	0.87 (0.82-0.92)*	0.92 (0.87-0.97)*
Semi- rural	0.89 (0.87-0.92)*	0.89 (0.87-0.91)*	0.89 (0.82-0.97)*	0.92 (0.85-1.01)
Rural	0.78 (0.77-0.80)*	0.80 (0.79-0.82)*	0.93 (0.86-0.99)*	1.01 (0.94-1.08)
**Personal income** ^1^				
Middle income	1.00	1.00	1.00	1.00
No income	3.47 (3.42-3.53)*	2.23 (2.20-2.26)*	8.86 (8.32-9.22)*	7.08 (6.72-7.46)*
Low income	1.85 (1.82-1.88)*	1.38 (1.36-1.40)*	2.82 (2.65-3.00)*	2.30 (2.17-2.44)*
High income	0.55 (0.54-0.56)*	0.62 (0.61-0.64)*	0.43 (0.39-0.46)*	0.47 (0.40-0.55)*
**Non-P-consultations with GP**				
Zero	1.00	1.00		
One	1.38 (1.36-1.40)*	1.21 (1.19-1.23)*	-	-
2-4	1.69 (1.67-1.72)*	1.42 (1.41-1.44)*	-	-
5+	2.33 (2.29-2.37)*	1.75 (1.72-1.77)*	-	-
**Non-P consultations at EPC services**				
Zero			1.00	1.00
One	-	-	3.27 (3.11-3.44)*	3.41 (3.24-3.59)*
2+	-	-	11.64 (10.99-12.32)*	10.88 (10.32-11.48)*

^1^20+ years only.

*p < 0.05.

For EPC P-consultations, a similar pattern to that of GP P-consultations was found for marital status, income and use of EPC services for other reasons. Age was inversely related to use of EPC services; odds were lower for middle aged and older adults than for young adults. Women who lived in the most urban areas only had higher odds than those in suburban areas whilst men in urban areas had higher odds than men in all other areas.

### P-consultations for Norwegians and immigrants by country of origin

**Figure 1:**
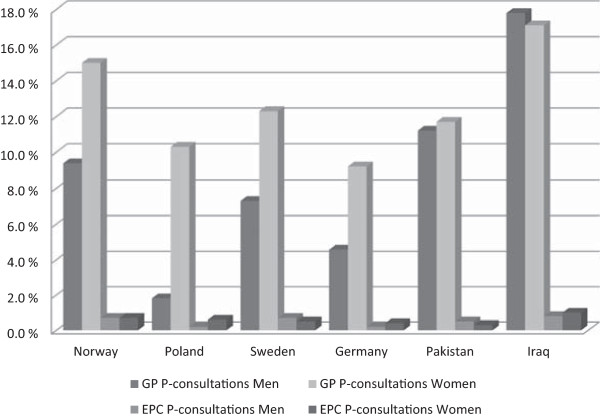
**Age-adjusted percentage of men and women with at least one GP P-consultation or EPC P-consultation by country of origin.**

Age-adjusted percentages of men and women, who had had a GP or an EPC P-consultation, by country of origin, are shown in Figure 1. It suggests that Polish men had the lowest GP P-consultation rate and Iraqi men had the highest. Pakistani and Iraqi men and Iraqi women appeared to have higher rates than their Norwegian counterparts. Within each country, Norwegian, Polish, Swedish and German women had higher rates of P-consultations than men while the differences between Pakistani and Iraqi men and women were very small.

After adjusting for age-group, marital status, place of residence, income and general GP use, men from Iraq had significantly higher odds of having had a GP P-consultation compared with Norwegian men. All other groups of men had significantly lower odds (see Table 3). All groups of immigrant women had significantly lower odds of having a GP P-consultation compared with Norwegian women, after adjusting for all background variables. Among both men and women, Polish immigrants had the lowest odds of having had a GP P-consultation compared with Norwegians. The odds for an EPC P-consultation were lower for all immigrant groups (except Swedes) compared with Norwegians.

**Table 3:** **Adjusted odds ratio (OR) and confidence intervals (CI) for GP and EPC P-consultations for men and women by country of origin** ^**1**^

	GP P-consultation		EPC P-consultation	
	Men Adj. OR (95% CI)	Women Adj. OR (95% CI)	Men Adj. OR (95% CI)	Women Adj. OR (95% CI)
Norway	1.00	1.00	1.00	1.00
Poland	0.14 (0.13-0.16)*	0.46 (0.43-0.50)*	0.42 (0.33-0.53)*	0.63 (0.48-0.82)*
Sweden	0.69 (0.65-0.74)*	0.80 (0.75-0.84)*	0.89 (0.72-1.11)	0.84 (0.66-1.07)
Germany	0.43 (0.39-0.58)*	0.56 (0.52-0.61)*	0.30 (0.18-0.48)*	0.60 (0.40-0.89)*
Pakistan	0.91 (0.85-0,98)*	0.54 (0.50-0.58)*	0.65 (0.48-0.87)*	0.26 (0.17-0.38)*
Iraq	1.20 (1.13-1.27)*	0.71 (0.66-0.77)*	0.68 (0.55-0.85)*	0.57 (0.43-0.74)*

^1^adjusted for age group, place of residence, marital status, income and use of GP/EPC services.

*p < 0.05.

### Importance of length of stay and reason for immigration

We also considered length of stay and reason for immigration for immigrants. Reason for immigration was not collected prior to 1990 and since Scandinavian immigrants are generally not required to provide this information, analysis only includes immigrants from Poland, Germany, Iraq and Pakistan who moved to Norway from 1990 onwards (44,673 men; 24,338 women). Table 4 shows that after adjusting for other background variables including country of origin, the odds of having a GP P-consultation were lower for immigrant women staying less than 12 years compared with those staying 12–18 years. For men, the odds were lower for immigrants staying 0.5-2 years and 6–11 years compared with those staying 12–18 years, but not for those staying 3–5 years. Men moving for work had lower odds of having had a P-consultation than those moving for family reunification, and men moving for protection had higher odds. Among women, only those moving for protection had higher odds of a GP P-consultation compared to those moving for family.

**Table 4:** **Adjusted odds ratio and confidence intervals for immigrant men and women by length of stay and reason for immigration** ^**1**^

	GP P-consultation		EPC P-consultation	
	Men Adj. OR (95% CI)	Women Adj. OR (95% CI)	Men Adj. OR (95% CI)	Women Adj. OR (95% CI)
**Length of stay**				
12-18 years	1.00	1.00	1.00	1.00
6-11 years	0.83 (0.73-0.95)*	0.85 (0.75-0.97)*	0.87 (0.54-1.43)	0.90 (0.54-1.48)
3-5 years	0.89 (0.74-1.05)	0.61 (0.52-0.71)*	0.90 (0.48-1.67)	0.42 (0.21-0.85)*
0.5-2 years	0.37 (0.30-0.44)*	0.29 (0.24-0.34)*	0.32 (0.17-0.64)*	0.18 (0.09-0.37)*
**Reason for immigration**				
Family	1.00	1.00	1.00	1.00
Work	0.47 (0.37-0.59)*	0.98 (0.83-1.16)	0.60 (0.27-1.32)	1.12 (0.55-2.31)
Protection	1.34 (1.12-1.61)*	1.38 (1.20-1.59)*	1.56 (0.79-3.06)	0.87 (0.50-1.53)
Other	0.88 (0.67-1.17)	0.92 (0.74-1.16)	0.60 (0.17-2.10)	1.14 (0.46-2.84)

^1^adjusted for country, length of stay and reason for immigration, plus age group, marital status, income, use of GP/EPC services (place of residence was not significant).

*p < 0.05.

Reason for immigration was not significantly related to odds of EPC P-consultations for men or women. The odds of an EPC P-consultation however, was significantly lower for men who had lived in Norway for less than 2 years, and women less than 6 years compared with immigrants living in Norway 12–18 years.

## Discussion

Overall, immigrants are less likely to use a GP or EPC services for mental health problems compared to Norwegians. As has previously been found in relation to general health [6, 13], we observed large variation across the different immigrant groups. As such, immigrants cannot be considered as a homogeneous group. Variations may reflect a combination of differences in mental health, differences in help-seeking, difficulties in accessing care or differences in understandings of, and appropriate treatment for, mental health. Further, the study also highlights the importance of considering men and women separately. While many background variables are related to P-consultations in similar ways for men and women, country of origin is not. Rates of GP P-consultations for Norwegians, Poles, Swedes and Germans are substantially higher among women than men but the difference is smaller among Pakistanis, and for Iraqis the rate is slightly higher among men.

Iraqi men were the only group with higher odds of a GP P-consultation than Norwegians after adjusting for background variables. Pre-migration experiences such as persecution, imprisonment and torture increase the risk of mental health problems, post-migration [16]. Immigrants from high conflict areas such as Iraq are likely to have had traumatic experiences and are thus at higher risk of mental health problems. In support of this, we also found that men and women who had moved to Norway for protection were more likely to have had a P-consultation with a GP than men and women moving for family reunification. A Norwegian study suggested that immigrant women from Middle Eastern countries including Iraq, scored highest on symptoms of distress of all other groups, and higher than immigrant men from the same area [17]. Yet, after accounting for background variables, we found that Iraqi women were actually less likely than Norwegian women to have had a P-consultation. This mismatch may suggest a difference in the way Iraqi men and women express their difficulties, or differences in help-seeking behaviour. Women may experience more barriers than men.

Another interesting finding is that although previous research suggests that Pakistanis report more mental health problems than Norwegians [18], they were less likely to have consulted with a GP or with EPC services in relation to a mental health problem. Pakistanis may also experience difficulties in accessing care. As well as language difficulties and lack of knowledge about the available services, different cultural understandings of mental health may make available services inappropriate for some immigrant groups [19, 20]. Among Pakistani women, fear of stigma is also a significant barrier to help seeking [21]. Stigma may contribute to the somatization of mental health problems which is common among this group, making the detection of mental health problems more difficult for a GP [22].

Polish immigrants had the lowest odds of a GP P-consultation for both men and women. A recent report however, suggests that Poles often report travelling home to use health care services there instead of in Norway [23]. This may also apply to other immigrant groups and explain some of the lower rates. It is unlikely however, to account for the seven-fold difference in Norwegian and Polish men’s GP P-consultations. While Polish immigrants experience some barriers in accessing health care in Norway [23], the lower rate may also be a reflection of the healthy migrant effect; suggesting that those who move to another country are on average healthier and more resourceful than individuals who do not migrate [24]. Indeed, the same report found that Poles living in Norway described being in a positive emotional state.

The healthy migrant effect has been shown to diminish through time though [24]. In line with this, we found longer length of stay to be associated with higher odds of a P-consultation. Since Polish immigrants have a shorter average length of stay than the other groups (they are mostly a recently arrived group), they may still be ‘healthy immigrants’ who experience significant mental health advantages compared with native Norwegians and other immigrant groups. Polish immigrants, as well as Germans and Swedes who also had lower rates, are predominately working immigrants. It may be that the healthy migrant effect applies to working migrants in particular. In further support of this, men moving for work were less likely to have had a GP P-consultation than men moving for family reunification, even after accounting for country of origin. Interestingly, there was no difference for women.

The current study distinguished between EPC and GP P-consultations. Previous research suggests that immigrants use of EPC services for all health problems declines with length of stay [6]. The authors suggested that this was because immigrants take time to understand the Norwegian health service and often use emergency care for non-urgent purposes, when an appointment with a GP would be suitable. However, the current study suggests that this is not the case in relation to mental health problems; as with GP P-consultations, the likelihood of having an EPC P-consultation increased with increased length of stay. This does not suggest that immigrants gradually switch from using EPC inappropriately to using GP services for mental health problems. Further, few individuals used EPC services for P-consultations exclusively. Thus EPC services appear to be used in addition to, and not as an alternative for, a GP. This is an important and encouraging observation since a regular GP, who has access to more background and contextual information, is in a better position to detect and treat mental health problems than an attending doctor at EPC services, who has limited information about a patient.

The data used in this study benefits from having nationwide coverage, overcoming issues such as representativeness and self-selection biases typically associated with survey data. There are however, limitations associated with the use of register data for research purposes. We are restricted by the variables we can investigate; other potential explanatory variables, including actual mental health status, would have been important to consider. As a result, we are only able to speculate as to why different immigrant groups have lower rates of service use than native Norwegians. Another factor that may contribute to lower rates is unregistered re-migration. Not all immigrants will de-register upon leaving Norway. The salmon bias hypothesis supposes that immigrants return to their home country when they become sick [25]. Thus, an overestimation of the absolute number of immigrants in Norway will result in an underestimation of percentage of immigrants with a P-consultation. Another limitation is our measure of income level. This may not be an accurate reflection of socioeconomic status, since we only have information on personal income and not household income. Despite this, the relationship found between income and mental health supports previous research [26].

## Conclusions

This study shows that use of primary health care services for mental health problems varies among immigrant groups in Norway. While some groups of immigrants may have lower rates of mental health problems, others may experience barriers to seeking help. Taken together with previous findings, the results suggest that Iraqi women and Pakistanis in particular may be groups that experience barriers in accessing care. Further investigation however, is required to determine this. It is important understand the barriers that immigrants in Norway may face, and what measures can be taken to reduce these. Consideration should be given to men and women separately, since patterns of service use can be quite different.

## Abbreviations

PHC: Primary health care services
EPC: Emergency primary care
GP: General practitioner
P-diagnosis: Psychiatric diagnosis
P-consultation: Consultation involving a psychiatric diagnosis.

## References

[1] Calderon-Larranaga A, Gimeno-Feliu LA, Macipe-Costa R, Poblador-Plou B, Bordonaba-Bosque D, Prados-Torres A (2011). Primary care utilisation patterns among an urban immigrant population in the Spanish National Health System. BMC Public Health.

[2] Dyhr L, Andersen JS, Engholm G (2007). The pattern of contact with general practice and casualty departments of immigrants and non-immigrants in Copenhagen, Denmark. Dan Med Bull.

[3] Sandvik H, Hunskaar S, Diaz E (2012). Immigrants’ use of emergency primary health care in Norway: a registry-based observational study. BMC Health Serv Res.

[4] Uiters E, Deville W, Foets M, Spreeuwenberg P, Groenewegen PP (2009). Differences between immigrant and non-immigrant groups in the use of primary medical care; a systematic review. BMC Health Serv Res.

[5] Nielsen SS, Krasnik A, Rosano A (2009). Registry data for cross-country comparisons of migrants’ healthcare utilization in the EU: a survey study of availability and content. BMC Health Serv Res.

[6] Goth US, Godager G (2012). Use of primary care emergency services in Norway: impact of birth country and duration of residence. Health Econ.

[7] Norredam M, Krasnik A, Sorensen TM, Keiding N, Michaelsen JJ, Nielsen AS (2004). Emergency room utilization in Copenhagen: a comparison of immigrant groups and Danish-born residents. Scand J Public Health.

[8] Rue M, Cabre X, Soler-Gonzalez J, Bosch A, Almirall M, Serna MC (2008). Emergency hospital services utilization in Lleida (Spain): a cross-sectional study of immigrant and Spanish-born populations. BMC Health Serv Res.

[9] Sundquist J (1993). Ethnicity as a risk factor for consultations in primary health care and out-patient care. Scand J Prim Health Care.

[10] Ku L, Matani S (2001). Left out: immigrants’ access to health care and insurance. Health Aff.

[11] Blom S (2008). Immigrants’ Health 2005/2006.

[12] Stronks K, Ravelli AC, Reijneveld SA (2001). Immigrants in the Netherlands: equal access for equal needs?. J Epidemiol Community Health.

[13] Nielsen SS, Hempler NF, Waldorff FB, Kreiner S, Krasnik A (2012). Is there equity in use of healthcare services among immigrants, their descendents, and ethnic Danes?. Scand J Public Health.

[14] Sanz B, Regidor E, Galindo S, Pascual C, Lostao L, Díaz J, Sánchez E (2011). Pattern of health services use by immigrants from different regions of the world residing in Spain. Int J Public Health.

[15] Statistics Norway (2008). Immigration Category 2008.

[16] Lien L, Thapa SB, Rove JA, Kumar B, Hauff E (2010). Premigration traumatic events and psychological distress among five immigrant groups: results from a cross-sectional study in Oslo, Norway. Int J Ment Health.

[17] Thapa S, Hauff E (2005). Gender differences in factors associated with psychological distress among immigrants from low- and middle-income countries. Soc Psychiatry Psychiatr Epidemiol.

[18] Syed HR, Dalgard OS, Dalen I, Claussen B, Hussain A, Selmer R, Ahlberg N (2006). Psychosocial factors and distress: a comparison between ethnic Norwegians and ethnic Pakistanis in Oslo, Norway. BMC Public Health.

[19] Li HZ, Browne AJ (2000). Defining mental illness and accessing mental health services: perspectives of Asian Canadians. Can J Commun Ment Health.

[20] Scheppers E, Van Dongen E, Dekker J, Geertzen J, Dekker J (2006). Potential barriers to the use of health services among ethnic minorities: a review. Fam Pract.

[21] Gilbert P, Gilbert J, Sanghera J (2004). A focus group exploration of the impact of izzat, shame, subordination and entrapment on mental health and service use in South Asian women living in Derby. Ment Health Relig Cult.

[22] Tabassum R, Macaskill A, Ahmad I (2000). Attitudes towards mental health in an Urban Pakistani community in the United Kingdom. Int J Soc Psychiatr.

[23] Czapka EA (2010). The Health of Polish Labour Immigrants in Norway – a Research Report.

[24] Lou Y, Beaujot R (2005). What happens to the ‘Healthy immigrant effect’: the mental health of immigrants to Canada. PSC Discuss Pap Ser.

[25] Abraído-Lanza AF, Dohrenwend BP, Ng-Mak DS, Turner JB (1999). The Latino mortality paradox: a test of the “salmon bias” and healthy migrant hypotheses. Am J Public Health.

[26] Jacobi F, Höfler M, Siegert J, Mack S, Gerschler A, Scholl L, Busch MA, Hapke U, Maske U, Seiffert I, Gaebel W, Maier W, Wagner M, Zielasek J, Wittchen HU (2014). Twelve-month prevalence, comorbidity and correlates of mental disorders in Germany: the Mental Health Module of the German Health Interview and Examination Survey for Adults (DEGS1-MH). Int J Methods Psychiatr Res.

[CR27] The pre-publication history for this paper can be accessed here: http://www.biomedcentral.com/1472-6963/14/341/prepub

